# The possible correlation between miR-762, Hippo signaling pathway, TWIST1, and SMAD3 in lung cancer and chronic inflammatory diseases

**DOI:** 10.1038/s41598-024-58704-5

**Published:** 2024-04-08

**Authors:** Neveen A. Hussein, Samia A. Ebid, Mohammad A. Ahmad, Gamal E. Khedr, Dina M. Saad

**Affiliations:** 1 Applied Medical Chemistry Department, Medical Research Institute, Alexandria University, Alexandria, Egypt; 2 Clinical Pathology Department, Military Medical Academy, Cairo, Egypt; 3 Clinical Pathology Department, Tanta Cancer Center, Tanta, Egypt

**Keywords:** MiR-762, Hippo signaling pathway, TWIST1, SMAD3, Lung Cancer, Biochemistry, Cancer, Molecular biology

## Abstract

MicroRNAs are small RNA molecules that have a significant role in translational repression and gene silencing through binding to downstream target mRNAs. MiR-762 can stimulate the proliferation and metastasis of various types of cancer. Hippo pathway is one of the pathways that regulate tissue development and carcinogenesis. Dysregulation of this pathway plays a vital role in the progression of cancer. This study aimed to evaluate the possible correlation between miR-762, the Hippo signaling pathway, TWIST1, and SMAD3 in patients with lung cancer, as well as patients with chronic inflammatory diseases. The relative expression of miR-762, MST1, LATS2, YAP, TWIST1, and SMAD3 was determined in 50 lung cancer patients, 30 patients with chronic inflammatory diseases, and 20 healthy volunteers by real-time PCR. The levels of YAP protein and neuron-specific enolase were estimated by ELISA and electrochemiluminescence immunoassay, respectively. Compared to the control group, miR-762, YAP, TWIST1, and SMAD3 expression were significantly upregulated in lung cancer patients and chronic inflammatory patients, except SMAD3 was significantly downregulated in chronic inflammatory patients. MST1, LATS2, and YAP protein were significantly downregulated in all patients. MiR-762 has a significant negative correlation with MST1, LATS2, and YAP protein in lung cancer patients and with MST1 and LATS2 in chronic inflammatory patients. MiR-762 may be involved in the induction of malignant behaviors in lung cancer through suppression of the Hippo pathway. MiR-762, MST1, LATS2, YAP mRNA and protein, TWIST1, and SMAD3 may be effective diagnostic biomarkers in both lung cancer patients and chronic inflammatory patients. High YAP, TWIST1, SMA3 expression, and NSE level are associated with a favorable prognosis for lung cancer.

## Introduction

Lung cancer continues to be a significant burden even with recent advancements in cancer diagnosis techniques. Lung cancer ranks among the worst cancers, accounting for 18.4% of all cancer-related deaths worldwide. In Egypt, lung cancer is the fifth and ninth most prevalent cancer in men and women, respectively and accounting for 5.0–7.0% of all cancer cases^1^. The most significant risk factor for all forms of lung cancer is smoking. Concomitant exposure to gases (asbestos or radon) or chronic lung disease may increase the risk of lung cancer^2^.

MicroRNAs (miRNAs), a varied class of endogenous noncoding RNAs, are essential for various biological processes, including immune responses, metabolism, apoptosis, cell proliferation, and migration^3^. Furthermore, they have been linked to the initiation, progression, and metastasis of different types of cancer by functioning as tumor suppressors or oncogenes. MiRNAs pair with the 3' untranslated region to control the expression of other types of genes at the post-transcriptional level^4^. One of the main factors contributing to the emergence and progression of numerous diseases, from inflammation to cancer, is miRNA dysfunction. MiR-762 expression was upregulated in numerous malignancies, including ovarian cancer^5^, gastric cancer^6^, and breast cancer, where it could promote the growth and spread of cancer cells^7^.

The Hippo signaling pathway is involved in cell regeneration, tissue repair, apoptosis, and proliferation. A disrupted Hippo pathway plays a role in the initiation and spread of cancer. After the Hippo pathway is trigged, activated mammalian sterile 20-like kinase1/2 (MST1/2) and WW45 phosphorylate large tumor suppressor kinases (LATS1/2), which causes Yes-associated protein (YAP) to be phosphorylated^8^. YAP was phosphorylated by this kinase cascade and rendered inactive through either proteasomal degradation or cytoplasmic retention. As a result, YAP cannot translocate to the nucleus or interact with TEA domain family members 1–4 (TEAD). It has been suggested that the YAP gene is a potential oncogene that may be directly linked to the development and progression of various types of cancer^9^.

TWIST1, a transcription factor belonging to the basic helix-loop-helix family, performs a variety of roles linked to the progression of tumors and fibrotic disorders including liver fibrosis^10,11^. In esophageal squamous cell carcinoma (ESCC) cell lines, TWIST1 is a crucial transcription factor that enhances the epithelial-mesenchymal transition (EMT) by downregulating E-cadherin and upregulating mesenchymal genes such as N-cadherin, ZEB2, vimentin, and fibronectin. Furthermore, TWIST1 suppresses apoptosis by downregulating Bax and upregulating Bcl-2^12^.

SMAD3, a crucial transcription factor for TGF-ꞵ signaling, is composed of a linker region, an N-terminal MH1 domain that interacts with the SMAD Binding Element (SBE), and a C-terminal MH2 domain that interacts with other proteins^13^. In canonical TGF-ꞵ signaling, activation of transforming growth factor beta receptor 1 (TGFBR1) phosphorylates SMAD2 and SMAD3, allowing heterodimerization with SMAD4. SMAD2-SMAD4 or SMAD3-SMAD4 complexes subsequently translocate to the nucleus to regulate activities such as apoptosis, proliferation, cell migration, and invasion in addition to controlling gene expression^14^.

Accordingly, this study aimed to evaluate the possible correlations between miR-762, Hippo signaling pathway, TWIST1, and SMAD3 in patients with chronic inflammatory diseases as well as lung cancer patients.

## Subjects and methods

Fifty newly diagnosed lung cancer patients (Tanta Cancer Center, Egypt), 30 non-cancer patients with chronic inflammatory diseases as obstructive pulmonary diseases, and asthma (Sadr El Mamoura Hospital, Alexandria), and 20 apparently healthy volunteers were enrolled in this study.

Participants with tuberculosis, pneumonia, and pyogenic lung abscesses were excluded. For lung cancer patients, the following tests were performed: chest x-rays and CT scans; routine laboratory tests; preoperative fine needle aspiration cytology of the lung mass to determine the pathological diagnosis.

Blood samples were taken from all patients before any treatment and from controls. These samples were used for the quantification of miR-762, MST1, LATS2, YAP, TWIST1, SMAD3 expression by real-time PCR, YAP protein level (phospho Ser 127) by ELISA, as well as neuron-specific enolase level (NSE) by electrochemiluminescence immunoassay.

### Expression profiles of miR-762, MST1, LATS2, YAP, TWIST1, SMAD3.

Total RNA was extracted using miRNeasy Mini Kit (Qiagen, Germany). The RNA concentrations were confirmed in each sample using Nanodrop spectrophotometer (Thermo Fisher Scientific). RevertAid™ First Strand cDNA Synthesis Kit (Thermo Fisher Scientific) was used to synthesize cDNA of MST1, LATS2, YAP, TWIST1, and SMAD3 genes. MiRCURY LNA RT Kit (Qiagen, Germany) for miR-762 reverse transcription.

The profile of MST1, LATS2, YAP, TWIST1, SMAD3 and GAPDH was determined using Maxima SYBR Green qPCR Master Mix (Thermo Scientific) under the following condition: 95 °C for 10 min, then 40 cycles at 95 °C for 15 s, 60 °C for 30 s, and 72 °C at 30 s. The profile of miR-762 and housekeeping gene U6 was determined using miRCURY SYBR Green Master Mix (Qiagen) under the following condition: 95 °C for 2 min, then 40 cycles at 95 °C for 10 s, annealing/extension 56 °C for 60 s. The relative quantitation of target genes was expressed as 2^−ΔΔCt^. The primer sequences (Invitrogen, Life Technologies) are shown in Table 1. For hsa-miR-762 primer (Catalog No: 339306).

**Table 1:** Primer sequences of miR-762, MST1, LATS2, YAP, TWIST1, SMAD3, GAPDH, and U6.

Genes	Primer sequences
hsa-miR-762	F 5′-GGGGCTGGGGCCGGGG-3′ R 5′-GAACATGTCTGCGTATCTC-3′
MST-1	F 5′-CTGTGTAGCAGACATCTGGTCC-3′ R 5′-CTGGTTTTCGGAATGTGGGAGG-3′
LATS2	F 5′-GTTCTTCATGGAGCAGCACGTG-3′ R 5′-CTGGTAGAGGATCTTCCGCATC-3′
YAP	F 5′-TGTCCCAGATGAACGTCACAGC-3′ R 5′-TGGTGGCTGTTTCACTGGAGCA-3′
TWIST1	F 5′-GCCAGGTACATCGACTTCCTCT-3′ R 5′-TCCATCCTCCAGACCGAGAAGG-3′
SMAD3	F 5′-TGAGGCTGTCTACCAGTTGACC-3′ R 5′-GTGAGGACCTTGTCAAGCCACT-3′
GAPDH	F 5′-GTCTCCTCTGACTTCAACAGCG-3′ R 5′-ACCACCCTGTTGCTGTAGCCAA-3′
U6	F 5′-CTCGCTTCGGCAGCACAT-3′ R 5′-TTTGCGTGTCATCCTTGCG-3′

### YAP and NSE level

The YAP protein (phospho Ser 127) (pg/ml) was determined by enzyme-linked immunosorbent assay (ELISA). The stop solution changes the color from blue to yellow and the intensity of the formed color was measured at 450 nm. Serum NSE (ng/ml) was determined by electrochemiluminescence immunoassay (ECLIA, Elecsys NSE, Roche Diagnostics) on the cobas-e immunoassay analyzer. The identification of NSE, hinges on the interplay between antibodies and the specific target molecule.

### Statistical analyses

IBM SPSS software package version 20.0*. *(Armonk, NY: IBM Corp) and MedCalc software version 20.100 used to analyze the data. For normally distributed quantitative variables, one-way analysis of variance (ANOVA) was used to compare between more than two groups, and the Post Hoc test (Tukey) was used for pairwise comparisons. For abnormally distributed quantitative variables, Kruskal Wallis test was used to compare between more than two studied groups and Post Hoc (Dunn's multiple comparisons test) for pairwise comparisons. Student t-test and​ Mann Whitney test were used to compare between two studied groups for normally and abnormally distributed quantitative variables, respectively. Spearman coefficient was used for correlation. The area under the receiver operating characteristic curve (ROC) denotes the diagnostic performance of the test. Kaplan–Meier Survival curve was used for the significant relation with progression free survival and overall survival.

### Ethics approval and consent to participate

The Ethics Committee of the Medical Research Institute, Alexandria University gave its approval to this study (E/C. S/N. T67/2020),﻿ and by the Declaration of Helsinki. All participants provided their informed consent.

## Results

Table 2 shows the characteristics of 20 controls (12 male, 8 female), 30 chronic inflammatory patients (21 male, 9 female), and 50 lung cancer patients (44 male, 6 female). Out of 50 lung cancer patients, 7 had grade I, 20 had grade II, and 23 had grade III. Regarding stage, 2 had stage II, 8 had stage III, and 40 had stage IV. For chronic inflammatory patients, 20 were smokers and all had NSE ˂ 16.3 ng/ml.

**Table 2:** Characteristics of control, chronic inflammatory patients, and lung cancer patients.

	Control n (%)	Chronic inflammatory patients n (%)	Lung cancer patients n (%)
Age (years)			
< 40	1 (5)		6 (12)
≥ 40	19 (95)	30 (100)	44 (88)
Gender			
Male	12 (60)	21 (70)	44 (88)
Female	8 (40)	9 (30)	6 (12)
Family history			
Negative			36 (72)
Positive			14 (28)
Smoking			
Yes	12 (60)	20 (66.7)	44 (88)
No	8 (40)	10 (33.3)	6 (12)
Histopathology of lung cancer			
NSCLC			39 (78)
SCLC			11 (22)
Tumor size			
< 5			16 (32)
> 5			34 (68)
Pathological grade			
I			7 (14)
II			20 (40)
III			23 (46)
Stage			
II			2 (4)
III			8 (16)
IV			40 (80)
Lymph node metastasis			
Negative			8 (16)
Positive			42 (84)
CEA (ng/ml)			
< 5	20 (100)	26 (86.7)	15 (30)
> 5		4 (13.3)	35 (70)
NSE (ng/ml)			
< 16.3	20 (100)	30 (100)	14 (28)
> 16.3			36 (72)

### MiR-762, MST1, LATS2, YAP gene, YAP protein, TWIST1, SMAD3 and NSE profiles and correlations

**Figure 1:**
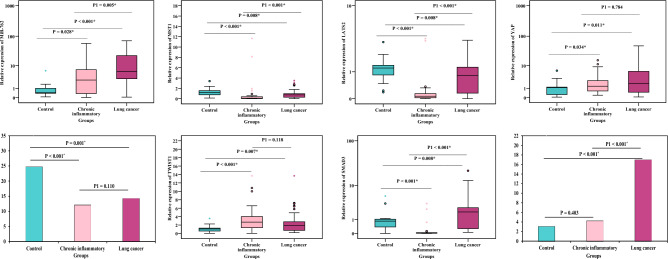
MiR-762, MST1, LATS2, YAP gene, YAP protein (pg/ml), TWIST1, SMAD3, and NSE (ng/ml) in control, chronic inflammatory and lung cancer groups.

Compared to the control group, the expression of miR-762, YAP, TWIST1, and SMAD3 were significantly upregulated in lung cancer patients (*P* = ˂ 0.001, 0.011, 0.007, 0.008, respectively) and chronic inflammatory patients (*P* = 0.028, 0.034, ˂ 0.001 respectively) except SMAD3 was significantly downregulated in chronic inflammatory patients (*P* ˂ 0.001). On the other hand, the expression of MST1, LATS2 and the concentration of YAP protein were significantly downregulated in lung cancer patients (*P* = 0.008, 0.008, 0.001, respectively) as well as chronic inflammatory patients (*P* ˂ 0.001). All studied parameters in lung cancer patients had significant difference compared to the corresponding values in chronic inflammatory patients except YAP expression, YAP protein, and TWIST1 (*P* = 0.784, 0.110, 0.118, respectively). The mean value of NSE was significantly increased in lung cancer patients compared to control group and chronic inflammatory patients (*P* ˂ 0.001) (Fig. 1).

**Figure 2:**
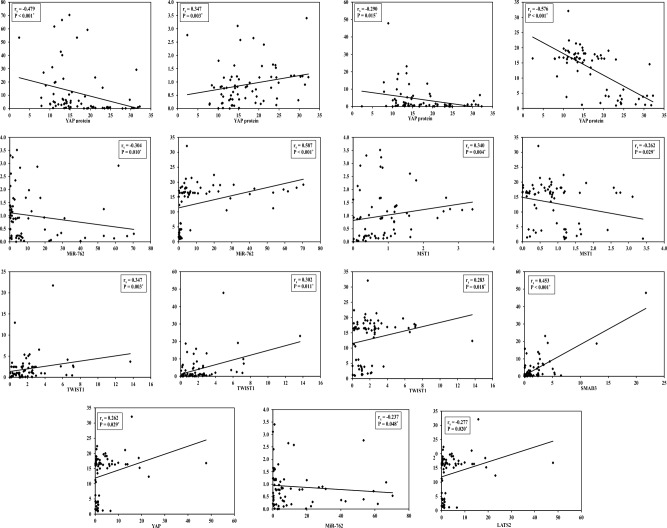
Correlations between miR-762, MST1, LATS2, YAP gene, YAP protein, TWIST1, SMAD3, and NSE in lung cancer patients using Spearman's coefficient test.

In chronic inflammatory patients, miR-762 was significantly negatively correlated with MST1 and LATS2 (*P* = 0.042, 0.045, respectively). There were significant positive correlations between each of MST1, LATS2, YAP protein, and SMAD3 (*P* = ˂ 0.001, 0.004, ˂ 0.001, ˂ 0.001, ˂ 0.001, 0.005, respectively). The expression of TWIST1 was correlated with YAP gene and protein (*P* = 0.009, and 0.001, respectively). In lung cancer patients, there were significant negative correlations between miR-762 and each of MST1, LATS2, and YAP protein (*P* = 0.048, 0.010, ˂ 0.001, respectively). Also, between YAP expression and YAP protein (*P* = 0.015). On the other hand, there were significant positive correlations between MST1 and each of LATS2, and YAP protein (*P* = 0.004, 0.003, respectively). Also, between each of YAP, TWIST1, and SMAD3 expression (*P* = 0.011, ˂ 0.001, and 0.003, respectively). NSE significantly correlated with all studied parameters except with SMAD3 (*P* = 0.199). In both chronic inflammatory patients and lung cancer patients CEA not correlated with all studied parameters (*P* ˃ 0.05) (Fig. 2, Supplementary Tables 1, 2).

### Relation of miR-762, MST1, LATS2, YAP gene and protein, TWIST1, SMAD3, and NSE with characteristics of the patients

**Figure 3:**
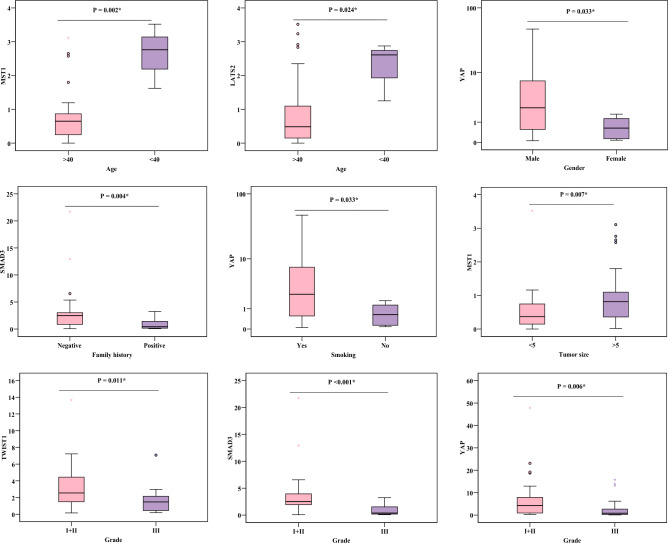
Relation between studied parameters and clinical characteristics of lung cancer patients.

No significant relation between all studied parameters and characteristics of chronic inflammatory patients (gender, smoking). On the other hand, significant relations were observed between some of the studied parameters and the characteristics of lung cancer patients. MST1 was related to age (*P* = 0.002) and tumor size (*P* = 0.007), YAP expression was related to gender, smoking, and grade (*P* = 0.033, 0.033, 0.006, respectively), SMAD3 was related to family history and grade (*P* = 0.004, ˂ 0.001), LATS2 was related to age (*P* = 0.024). Finally, TWIST1 was related to grade (*P* = 0.011) (Fig. 3, Supplementary Table 3).

### ROC and Kaplan–Meier

**Figure 4:**
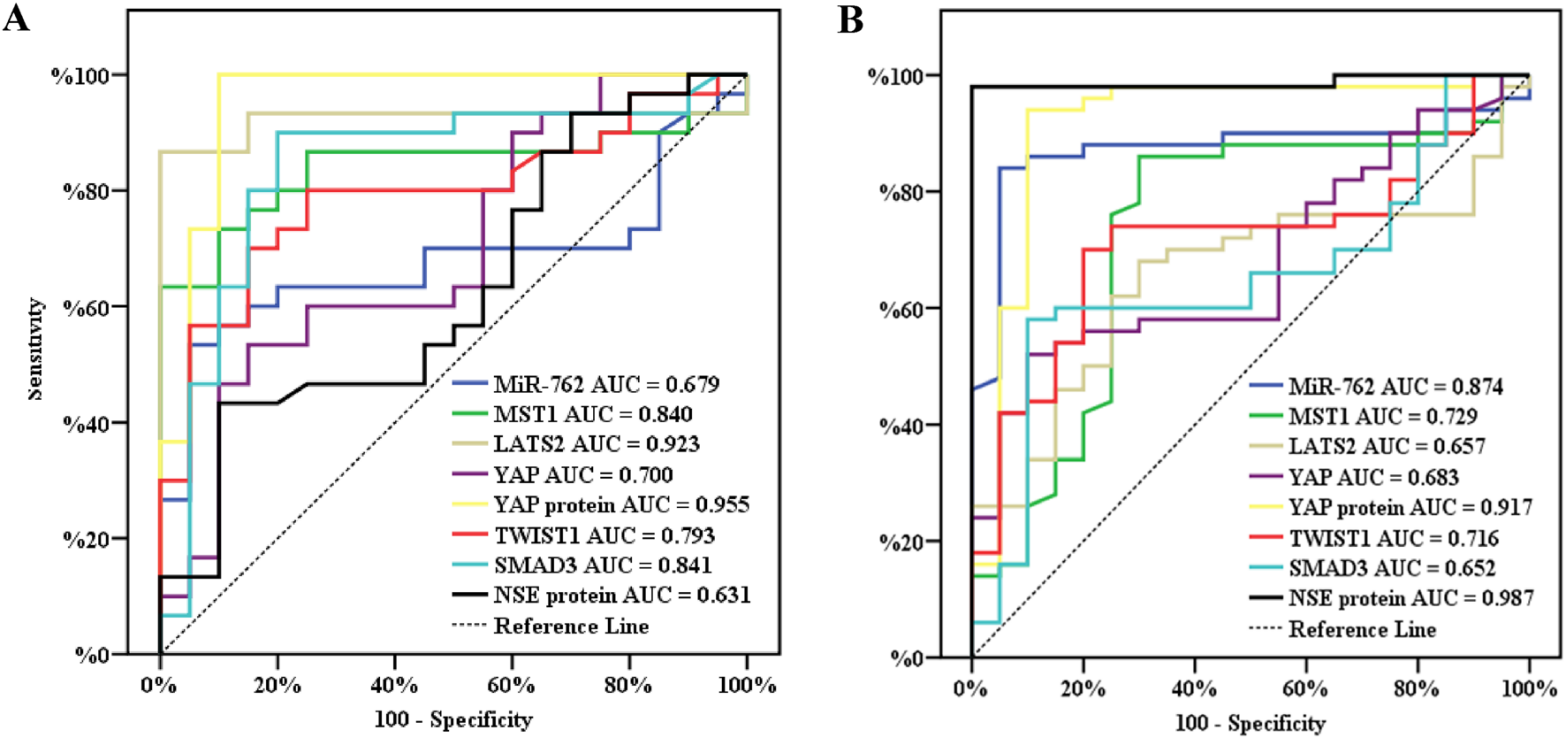
ROC curves for (**A**) chronic inflammatory patients, and (**B**) lung cancer patients.

The ROC curve was applied to compare the diagnostic values of miR-762, MST1, LATS2, YAP gene and protein, TWIST1, SMAD3 and NSE according to area under the curve (AUC). In chronic inflammatory patients, the AUC of studied parameters were (67.9, 84.0, 92.3, 70.0, 95.5, 79.3, 84.1, 63.1% respectively, *P* = 0.033, ˂0.001, ˂0.001, 0.017, ˂0.001, 0.001, ˂0.001, 0.120) with sensitivity (70.0, 86.7, 93.3, 63.3, 96.7, 80.0, 90.0, 56.7%, respectively) and specificity (55, 75, 85, 50, 90, 75, 80, 50%, respectively). In lung cancer patients, all these parameters had a higher AUC (87.4, 72.9, 65.7, 68.3, 91.7, 71.6, 65.2, 98.7%, respectively, *P* = ˂0.001, 0.003, 0.041, 0.018, ˂0.001, 0.005, 0.048, ˂0.001) with sensitivity (84, 86, 68, 80, 94, 70, 58, 98%, respectively) and specificity (95, 70, 70, 35, 90, 80, 90, 100%, respectively) (Table 3, Fig. 4).

**Table 3:** Diagnostic performance of studied parameters to discriminate chronic inflammatory patients (n = 30) and lung cancer patients (n = 50) from control (n = 20).

		Control	Patients	Sensitivity	Specificity	AUC	NPV	PPV	Accuracy	*P*
Chronic inflammatory patients										
MiR-762	≤ 0.46	11	9	70.0	55.0	67.9	55.0	70.0	64.0	0.033*
	> 0.46	9	21							
MST1	> 0.92	15	4	86.7	75.0	84.0	78.9	83.9	82.0	< 0.001*
	≤ 0.92	5	26							
LATS2	> 0.36	17	2	93.3	85.0	92.3	89.5	90.3	90.0	< 0.001*
	≤ 0.36	3	28							
YAP	≤ 1.12	10	11	63.3	50.0	70.0	47.6	65.5	58.0	0.017*
	> 1.12	10	19							
YAP protein	> 18.88	18	1	96.7	90.0	95.5	94.7	93.5	94.0	< 0.001*
	≤ 18.88	2	29							
TWIST1	≤ 1.167	15	6	80.0	75.0	79.3	71.4	82.8	78.0	0.001*
	> 1.167	5	24							
SMAD3	> 0.12	16	3	90.0	80.0	84.1	84.2	87.1	86.0	< 0.001*
	≤ 0.12	4	27							
NSE protein	≤ 3.05	10	13	56.7	50.0	63.1	43.5	63.0	54.0	0.120
	> 3.05	10	17							
Lung cancer patients										
MiR-762	≤ 1.75	19	8	84.0	95.0	87.4	70.4	97.7	87.14	< 0.001*
	> 1.75	1	42							
MST1	> 1.17	14	7	86.0	70.0	72.9	66.7	87.8	81.43	0.003*
	≤ 1.17	6	43							
LATS2	> 0.94	14	16	68.0	70.0	65.7	46.7	85.0	68.57	0.041*
	≤ 0.94	6	34							
YAP	≤ 0.45	7	10	80.0	35.0	68.3	41.2	75.5	67.14	0.018*
	> 0.45	13	40							
YAP protein	> 20.16	18	3	94.0	90.0	91.7	85.7	95.9	92.86	< 0.001*
	≤ 20.16	2	47							
TWIST1	≤ 1.38	16	15	70.0	80.0	71.6	51.6	89.7	72.86	0.005*
	> 1.38	4	35							
SMAD3	≤ 1.11	18	21	58.0	90.0	65.2	46.2	93.5	67.14	0.048*
	> 1.11	2	29							
NSE protein	≤ 8.38	20	3	98.0	100.0	98.7	100	93.9	95.71	< 0.001*
	> 8.38	0	47							

*AUC* Area Under the Curve; *NPV* Negative Predictive Value; *PPV* Positive Predictive Value.

*Statistically significant at *P* ≤ 0.05.

**Figure 5:**
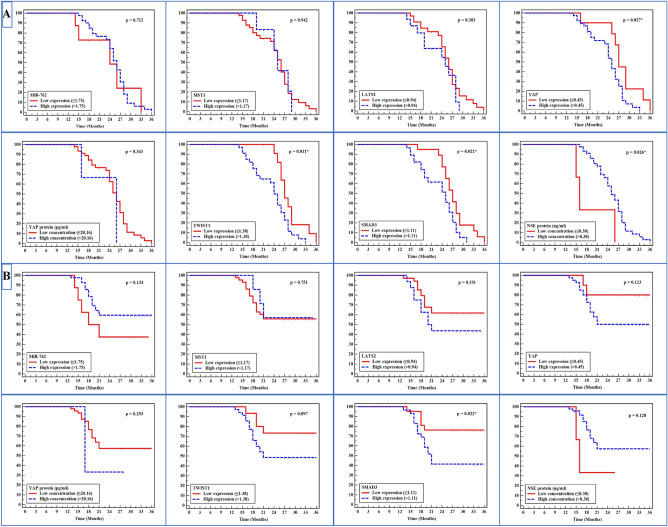
Kaplan Meier curves (**A**) DFS and (**B**) OS of miR-762, MST1, LATS2, YAP gene, YAP protein (pg/ml), TWIST1, SMAD3, and NSE (ng/ml) in lung cancer patients; *Statistically significant at *P* ≤ 0.05.

From the follow-up data of lung cancer patients, the survival curve analyses confirmed significant differences in Disease Free Survival (DFS) for patients with higher expression of YAP, TWIST1, SMAD3 and the level of NSE than those with lower expressions (*P* = 0.027, 0.011, 0.021, and 0.026, respectively). For Overall Survival (OS), SMAD3 expression had significant differences between higher and lower expression (*P* = 0.022) (Table 4, Fig. 5).

**Table 4:** Disease free survival and overall survival of all studied parameters in lung cancer patients.

		Lung cancer (n = 50)	Disease free survival				Overall survival			
			Metastatic n (%)	Mean	Log-rank		Death n (%)	Mean	Log-rank	
					χ^2^	*P*			χ^2^	*P*
MiR-762	≤ 1.75	8	5 (62.5)	24.11	0.136	0.712	5 (62.5)	23.63	2.241	0.134
	> 1.75	42	34 (81.0)	25.0			17 (40.5)	28.76		
MST1	≤ 1.17	43	34 (79.1)	24.73	0.005	0.942	19 (44.2)	27.84	0.101	0.751
	> 1.17	7	5 (71.4)	25.25			3 (42.9)	28.43		
LATS2	≤ 0.94	34	27 (79.4)	25.50	1.061	0.303	13 (38.2)	29.12	1.710	0.191
	> 0.94	16	12 (75.0)	23.29			9 (56.3)	25.19		
YAP	≤ 0.45	10	9 (90.0)	27.81	4.866	0.027*	2 (20.0)	32.30	2.380	0.123
	> 0.45	40	30 (75.0)	23.94			20 (50.0)	26.93		
YAP protein	≤ 20.16	47	37 (78.7)	24.97	0.901	0.343	20 (42.6)	28.30	1.308	0.253
	> 20.16	3	2 (66.7)	20.67			2 (66.7)	20.67		
TWIST1	≤ 1.378	15	11 (73.3)	28.27	6.502	0.011*	4 (26.7)	31.40	2.762	0.097
	> 1.378	35	28 (80.0)	23.30			18 (51.4)	26.54		
SMAD3	≤ 1.11	21	17 (81.0)	27.21	5.309	0.021*	5 (23.8)	31.57	5.265	0.022*
	> 1.11	29	22 (75.9)	23.01			17 (58.6)	25.41		
NSE (ng/ml)	≤ 8.38	3	3 (100.0)	19.00	4.966	0.026*	2 (66.7)	19.0	2.319	0.128
	> 8.38	47	36 (76.6)	25.15			20 (42.6)	28.36		

*Statistically significant at *P* ≤ 0.05.

## Discussion

The Hippo pathway is one of multiple pathways that regulate tissue development and carcinogenesis. Dysregulation of this pathway plays a vital role in the progression of numerous types of human cancers^15^. MiRNAs are essential for several biological processes because they regulate the expression of post-transcriptional genes. Expression of miRNAs is frequently tightly controlled. MiRNA dysfunction is one of the main factors contributing to the emergence and progression of numerous diseases from organ inflammation to cancer^16^.

MiR-762 functions mainly by regulating mRNA of several downstream genes, including RNase7, menin, suppression of tumorigenicity-2 (ST2), PH domain, and leucine-rich repeat protein phosphatase 2 (PHLPP2) and Forkhead box O4 (FOXO4)^5,17^. PHLPP2 plays an important role in tumor suppression and its down-regulation increases growth and migration of several cancers (HCC, CRC, gastric cancer, and ovarian cancer)^18,19^. As a phosphatase, PHLPP2 dephosphorylates protein kinase B (AKT) to decrease its activity. In gastric cancer, suppression of PHLPP2 leads to up-regulation of pAKT and the acceleration of EMT^20^. FOXO4 as a tumor suppressor induces apoptosis and prevents proliferation and invasion. Down-regulation of FOXO4 not only enhances the growth and metastasis of cervical cancer, but also promotes EMT^21^. Through modifying PHLPP2 and FOXO4, miR-762 expression may stimulate the AKT signalling pathway in head and neck squamous cell carcinoma and promote migration, proliferation, and EMT^17^. Additionally, miR-762 was predicted to have a binding site in 3′-UTR of menin where miR-762 could directly suppress the expression of menin^5^. Menin is a tumor suppressor gene that prevents the growth and occurrence of different types of tumors. Menin can decrease the nuclear accumulation and transcriptional activity of β-catenin^22^. β-Catenin is the most important molecule in the Wnt signaling pathway. Also, β-catenin can stimulus metastasis via affecting EMT and matrix metalloproteinases (MMPs), particularly β-catenin/MMP7 pathway. MiR-762 may stimulate the β-catenin/MMP7 pathway, which in turn may facilitate metastasis of ovarian cancer^5^. Previous studies indicated that miR-762 was significantly upregulated in NSCLC patients^23^, while PHLPP2 was downregulated to accelerate lung carcinogenesis by elevating inflammatory tumor necrosis factor alpha (TNFα)^24^. Furthermore, FOXO4 might prevent EMT in NSCLC^25^, and other tumor suppressor genes, such as menin, were downregulated^26^. Accordingly, miR-762 may exert its oncogenic effect through these tumor suppressor genes in lung cancer patients.

The present results revealed that the expression of MST1, LATS2 was significantly downregulated whereas the expression of YAP gene was significantly upregulated, and the level of phosphorylated YAP protein was significantly decreased in both chronic inflammatory patients as well as in lung cancer patients. Additionally, significant correlations were observed between most components of the Hippo pathway in all patients. Various tumors have been reported to exhibit down-regulation of MST1 expression^27,28^. As a tumor suppressor gene, MST1 makes cells extremely susceptible to death receptor-mediated apoptosis through accelerating caspase-3 activation, which in turn promotes apoptosis^29^. In pancreatic cancer, TNF receptor-associated factor 6 increased the degradation of MST1 via the ubiquitination degradation pathway, overexpressed YAP and regulated tumor cell growth and metastasis^30^. A previous study reported that α2β1 integrin may be an upstream negative regulator of the Hippo pathway in HCC. When α2β1 integrin binds to extracellular collagen, it becomes active and suppress MST1 and LATS1 kinase activity. YAP is released from the negative regulation of the Hippo core kinases and is transported into the nucleus to activate gene transcriptions for cell survival and proliferation. This helping cancer cells to overcome the cell–cell contact inhibition^31^.

Epigenetic inactivation of tumor suppressor genes is a primary mechanism for altered gene expression in tumors and has been commonly observed in human cancers. Mechanistically, hypermethylation of cytosine at CpG-rich sequences known as CpG islands, mediates the inactivation of tumor suppressor gene promoter regions^32^. Previous research found that decreased MST1 transcript expression is correlated with methylation of its promoter region^27^. Additionally, methylation alterations frequently inactivate LATS2, making the Hippo signaling pathway unable to control caner development^33^.

In a variety of cancer cells, YAP and TAZ are more active and strongly expressed. Dysregulation of the Hippo pathway in cancer cells may be caused by abnormally elevated levels of O-linked-N-acetylglucosaminylation (O-GlcNAcylation). YAP O-GlcNAcylation increases its activity by preventing the interaction between YAP and LATS1^34^. Activation of YAP / TAZ causes LATS2 to become more transcriptionally active, which in turn suppresses YAP/TAZ activity^9^. This process represents a negative feedback loop in Hippo pathway that contributes to maintaining homeostasis in terms of intracellular YAP / TAZ levels and activities. Therefore, it can be logically inferred that YAP O-GlcNAcylation reduces TAZ levels by promoting LATS2 activity. But TAZ remains high and LATS activity is suppressed in different cancer cells^35^. As a result, additional Hippo pathway components are O-GlcNAcylated, which disrupts the network structure of cancer cells. Defects in the Hippo pathway negative feedback loop resulting from LATS2 deletion, and an elevated intracellular O-GlcNAcylation level can trigger carcinogenesis. Thus, LATS2 O-GlcNAcylation may contribute to carcinogenesis by obstructing the negative feedback loop of Hippo pathway^36^.

As a result of genomic amplification, malignant cells produce an excessive amount of YAP, which overwhelms normal physiological regulating systems and causes aberrant cytoplasmic accumulation^37^. Once Hippo signaling is lost, YAP increased in the nucleus and becomes more active. YAP interacts with transcription factors, specially TEAD, to control the transcription of target genes by binding to distant enhancers and contacting their regulated promoters through DNA looping^38^. The Bcl-2 gene promoter has TEAD binding sites, so YAP may be recruited to the Bcl-2 promoter by binding with TEAD. By transcriptionally upregulating Bcl-2, YAP can suppress autophagy, and consequently accelerates CRC growth^39^.

The phosphorylation of YAP at Ser127 is commonly used as a marker for YAP inactivation^40^. YAP1 is regulated by nuclear Dbf2-related / LATS kinases, which facilitate its phosphorylation at Serine 127. This phosphorylation leads to the exclusion of YAP1 from the nucleus to the cytoplasm, where it binds to the 14–3-3 protein and undergoes degradation via the ubiquitin proteasome system. Accordingly, the transcription of pro-growth genes is repressed^41^. Akt kinase is another kinase that enhances the binding of 14–3-3 protein to YAP. Akt-induced YAP suppression results in the inhibition of transcription factors, including p53, a regulator of pro-apoptotic gene B-cell lymphoma-2 associated X protein. Therefore, phosphorylated inactive YAP inhibits the pro-apoptotic gene expression in response to cellular damage^42^.

The observed significant overexpression of TWIST1 and positive correlation with YAP1 in both chronic inflammatory patients and lung cancer patients may be attributed to forced expression of YAP1 that elevated TWIST1 mRNA and protein. The promoter region of the TWIST1 gene has a potential TEAD1-binding site, revealing that YAP1/TEAD1 may be able to control TWIST1 at the transcriptional level. In lung fibroblasts, YAP1 promotes fibrogenesis via the formation of the YAP1/TEAD complex, which in turn transcriptionally increases the expression of TWIST1^43^.

The present results showed that SMAD3 expression was significantly upregulated and correlated with YAP in lung cancer patients. SMAD3 accelerates the growth of lung cancer by affecting downstream factors. The RAB26 promoter is bound to SMAD3, which increases RAB26 expression and NSCLC growth^44^. Additionally, SMAD3 promotes the development of lung cancer by influencing lung adenocarcinoma-associated fibroblasts and tumor-associated fibroblasts, which are vital for the tumor microenvironment-driven development of cancer. Increased expression of SMAD3 stimulates fibroblast migration^45^. Besides, regardless the ability of YAP to bind TEAD, YAP interacts with SMAD3 to promotes the TGF-β/SMAD3 transcriptional activity, which in turn improving TGF-β ability to enhance lung metastasis. TGF-β/SMAD3 is essential for YAP driven lung metastasis as TGF-β promotes “EMT-like”, cell migration and invasion by upregulating the expression of MMP-2/MMP-9^46^.

Under physiological conditions, NSE expression is restricted to specific tissues. Elevated level of NSE has been identified in neurogenic and neuroendocrine cancers, and act as a marker to indicate neuroendocrine differentiation of tumor cells. NSE participates in the progression of cancer where upon stimulation, its cellular localization changes to the cell surface to activate survival-promoting pathways, much as it does in neurons, and encourage the migration of cancer cells^47^.

Inflammation is an adaptive reaction driven by stressful situations and plays a crucial role in cancer growth and progression^15^. The lung is continuously subjected to a variety of stresses, including air pollution, free radicals, and chemical irritants. MiRNAs are essential to protect the host from these external threats. Dysregulated miRNA profiles have been found in many lung disorders. The pathogenesis of various pulmonary diseases, including smoking-related diseases and cancer, is mostly driven by miRNA aberration^48^. The Hippo signaling pathway may be associated with pulmonary diseases when it is uncontrolled^49^. In general, dysregulated Hippo pathway is often observed in the development of various pulmonary disorders such as pulmonary fibrosis, inflammatory pulmonary diseases, and pulmonary arterial hypertension.

YAP is essential in the inflammation-induced cancer process since it can function as a transcriptional co-activator and interact with other transcription factors to influence the expression of inflammation-associated factors. On the other hand, YAP not only caused inflammation, but also decreased it based on inflammation-associated factors^50^. YAP significantly increases the expression of inflammatory factors, such as TNFα and IL-1β^51^.

Elevated expression of YAP stimulates transcription of TWIST1 gene, which in turn causes proliferation of fibroblasts, deposition of collagen, and transition of fibroblasts from a relatively static state to a pathogenic activate state. Consequently, pulmonary fibrosis is induced^43^. Significant down-regulation of SMAD3 in chronic inflammatory patients may be attributed to smoking. Exposure to cigarette smoke particles and condensation of these particles in the proximal airways enhance SMAD3 promoter methylation leading to SMAD3 suppression^52^.

The observed insignificant relations of studied parameters with the characteristics of chronic inflammatory patients and with most clinical characteristics of lung cancer patients may be attributed to gender effects and the small sample size. These results in line with previous studies^17,53,54^. This study reported significant correlations between miR-762 expression and some of the components of the Hippo pathway. These results are in consistent with previous studies that indicated that several miRNAs are negatively regulate Hippo tumor-suppressor signaling pathway and enhance tumor growth^55,56^. In addition to controlling cellular activity at baseline, miRNAs also control cell behavior in diseases and under different stress conditions. Moreover, miRNAs regulate almost all biological processes by acting as a web of mediators^48^. Since miR-762 repress the downstream PHLPP2 gene^43^ which in turn downregulate MST1 expression^57^. So, miR-762 may exert its effect on Hippo pathway through PHLPP2.

Significant correlations between some of the components of the Hippo pathway, TWIST1 and SMAD3 were also observed. Elevated YAP expression and/or activation have been described in several types of solid tumor and linked to poor prognosis. YAP functions as an oncogene by activating target genes that stimulate the proliferation, EMT, and metastases^9,46,58^. YAP binds to the Smad2/3-Smad4 complex to sustain its nuclear accumulation and stimulate the transcription of several genes^59^. Also, YAP can control the expression of TWIST1 by binding to TEAD1 in the TWIST1 promoter region^43^. TWIST1 is extensively expressed in cancer, and through controlling the TGF-β/SMAD3 signaling pathway, it promotes EMT and carcinogenesis^60^.

ROC curves proved that the viability of using all studied parameters as diagnostic markers for lung cancer patients. NSE protein is superior to YAP protein followed by miR-762, MST1, TWIST1, YAP gene, LATS2, and SMAD3 for the prediction of lung cancer. Also, all studied parameters can be used as biomarkers in chronic inflammatory patients except NSE. The survival analyses showed that higher SMAD3 expression was related to lung cancer patients' worse DFS and OS. Moreover, high expression of YAP gene, TWIST1, and concentration of NSE predicted poor survival in lung cancer patients.

## Conclusion

MiR-762 is upregulated and negatively correlated with the Hippo pathway in both lung cancer patients and chronic inflammatory patients. MiR-762 may be involved in the induction of malignant behaviors of lung cancer through suppression of Hippo pathway. MiR-762, MST1, LAT2, YAP mRNA and protein, TWIST1, SMAD3 may be effective diagnostic biomarkers in both lung cancer patients and chronic inflammatory patients. High YAP, TWIST1, SMA3 expression, and NSE level as effective molecular biomarkers is associated with a favorable prognosis of lung cancer.

### Supplementary Information

Supplementary Tables.

## Data Availability

All data analyzed during this study are included in the article.
