# Supplementary material for: The possible correlation between miR-762, Hippo signaling pathway, TWIST1, and SMAD3 in lung cancer and chronic inflammatory diseases
Source: Sci Rep. 2024 Apr 8;14:8246. doi: 10.1038/s41598-024-58704-5 (PMC11001855; doi:10.1038/s41598-024-58704-5)
Supplement: Supplementary file 1 — Supplementary Tables. [file 41598_2024_58704_MOESM1_ESM.docx]

Table 1: Correlations between miR-762, MST1, LATS2, YAP gene, YAP protein (pg/ml), TWIST1, SMAD3, and NSE (ng/ml) in chronic inflammatory patients and lung cancer patients.

|  | | **Chronic inflammatory patients** | | | | | | | |  | **Lung cancer patients** | | | | | | |
| --- | --- | --- | --- | --- | --- | --- | --- | --- | --- | --- | --- | --- | --- | --- | --- | --- | --- |
|  | | **MiR-762** | | **MST1** | **TWIST1** | **SMAD3** | **YAP** | **LATS2** | **NSE** |  | **MiR-762** | **MST1** | **TWIST1** | **SMAD3** | **YAP** | **LATS2** | **NSE** |
| **YAP  protein** | r_s_  P | | -0.215  0.133 | 0.364  0.009* | -0.461  0.001* | 0.390  0.005* | -0.240  0.093 | 0.510  <0.001* | -0.166  0.250 |  | -0.479  <0.001* | 0.347  0.003* | -0.100  0.412 | -0.226  0.060 | -0.290  0.015* | 0.141  0.244 | -0.576  <0.001* |
| **MiR-762** | r_s_  P | |  | -0.322  0.023* | 0.163  0.257 | -0.134  0.354 | 0.041  0.776 | -0.285  0.045* | 0.178  0.216 |  |  | -0.237  0.048* | 0.042  0.730 | 0.110  0.366 | 0.056  0.645 | -0.304  0.010* | 0.587  <0.001* |
| **MST1** | r_s_  P | |  |  | -0.152  0.292 | 0.705  <0.001* | 0.025  0.864 | 0.692  <0.001* | 0.047  0.744 |  |  |  | 0.042  0.731 | -0.061  0.617 | -0.055  0.649 | 0.340  0.004* | -0.262  0.029* |
| **TWIST1** | r_s_  P | |  |  |  | -0.110  0.447 | 0.365  0.009* | -0.180  0.212 | 0.199  0.165 |  |  |  |  | 0.347  0.003* | 0.302  0.011* | 0.135  0.266 | 0.283  0.018* |
| **SMAD3** | r_s_  P | |  |  |  |  | 0.113  0.436 | 0.757  <0.001* | -0.086  0.551 |  |  |  |  |  | 0.453  <0.001* | 0.212  0.078 | 0.155  0.199 |
| **YAP** | r_s_  P | |  |  |  |  |  | -0.036  0.805 | 0.045  0.759 |  |  |  |  |  |  | 0.216  0.073 | 0.262  0.029* |
| **LATS2** | r_s_  P | |  |  |  |  |  |  | -0.086  0.554 |  |  |  |  |  |  |  | -0.277  0.020* |

r_s_: Spearman coefficient *: Significant at P ≤ 0.05

Table 2: correlation of studied parameters with CEA in chronic

Inflammatory patients and lung cancer patients.

|  | **CEA** | | | |
| --- | --- | --- | --- | --- |
|  | **Chronic inflammatory patients** | | **Lung cancer patients** | |
|  | r_s_ | P | r_s_ | P |
| **MiR-762** | -0.220 | 0.244 | 0.016 | 0.914 |
| **MST1** | -0.001 | 0.996 | 0.018 | 0.904 |
| **LATS2** | -0.197 | 0.297 | 0.070 | 0.631 |
| **YAP** | -0.131 | 0.489 | -0.144 | 0.319 |
| **YAP protein** | -0.001 | 0.996 | 0.024 | 0.871 |
| **TWIST1** | -0.263 | 0.161 | -0.144 | 0.318 |
| **SMAD3** | -0.285 | 0.127 | -0.016 | 0.910 |
| **NSE** | -0.107 | 0.574 | 0.102 | 0.479 |

r_s_: Spearman coefficient *: Significant at P ≤ 0.05

Table 3: Relation of studied parameters with characteristics of chronic inflammatory patients and lung cancer patients.

|  | **n** | **MiR-762** | **MST1** | **LATS2** | **YAP** | **YAP protein** | **TWIST1** | **SMAD3** | **NSE protein** |
| --- | --- | --- | --- | --- | --- | --- | --- | --- | --- |
|  |  | **Chronic inflammatory patients (n = 30)** | | | | | | | |
| **Gender**  **Male**  **Female**  **U (P) or t (P)** | **21**  **9** | 9.11 ± 14.32  2.02 ± 2.71  U=58.0 (P=0.104) | 1.18 ± 2.99  0.29 ± 0.5  U=64.50(P=0.178) | 0.40 ± 1.06  0.12 ± 0.13  U=85.0 (P=0.689) | 2.81 ± 3.73  2.42 ± 3.41  U=91.0 (P=0.894) | 12.49 ± 4.24  10.94 ± 3.41  t=0.964(P=0.343) | 3.84 ± 3.61  2.66 ± 1.94  U=85.0 (P=0.689) | 0.30 ± 0.88  0.12 ± 0.24  U=88.0 (P=0.790) | 4.47 ± 2.83  3.49 ± 2.19  U=73.0 (P=0.349) |
| **Smoking**  **Yes**  **No**  **U (P) or t (P)** | **22**  **8** | 8.86 ± 14.03  1.83 ± 2.83  U=49.0 (P=0.070) | 1.13 ± 2.93  0.32 ± 0.53  U=66.0 (P=0.320) | 0.39 ± 1.04  0.12 ± 0.14  U=87.0 (P=0.982) | 2.71 ± 3.67  2.63 ± 3.58  U=85.0 (P=0.909) | 12.48 ± 4.14  10.78 ± 3.60  t=1.027(P=0.313) | 3.76 ± 3.54  2.72 ± 2.07  U=81.0 (P=0.765) | 0.28 ± 0.86  0.13 ± 0.25  U=84.0(P=0.872) | 4.36 ± 2.81  3.66 ± 2.27  U=74.0 (P=0.534) |
|  |  | **Lung cancer patients (n = 50)** | | | | | | | |
| **Gender**  **Male**  **Female**  **U(P) or t (P)** | **44**  **6** | 16.28 ± 19.83  12.77 ± 20.73  U=97.50(P=0.311) | 0.75 ± 0.68  1.50 ± 1.41  U=93.0 (P=0.258 | 0.83 ± 0.89  1.77 ± 1.17  U=68 (P=0.057) | 5.92 ± 8.76  0.73 ± 0.63  U= 61 (P=0.033*) | 14.06 ± 4.23  15.15 ± 7.03  t=0.546(P=0.588) | 2.60 ± 2.69  2.07 ± 0.50  U=123.50(P=0.805) | 2.57 ± 3.76  1.41 ± 1.16  U=112.0 (P=0.570) | 17.32 ± 3.28  14.11 ± 6.10  U=81.50 (P=0.134) |
| **Age**  **>40**  **<40**  **U(P) or t (P)** | **47**  **3** | 15.39 ± 19.49  23.22 ± 27.16  U=61.5 (P=0.728) | 0.72 ± 0.67  2.63 ± 0.96  U=5.0 (P=0.002*) | 0.86 ± 0.91  2.25 ± 0.87  U=17.0 (P=0.024) | 5.60 ± 8.57  0.62 ± 0.88  U=24.0 (P=0.059) | 14.26 ± 4.18  13.14 ± 10.36  t=0.185(P=0.870) | 2.58 ± 2.60  1.87 ± 0.69  U=68.0 (P=0.939) | 2.52 ± 3.66  0.96 ± 0.44  U=53.0 (P=0.507) | 16.97 ± 3.91  16.33 ± 0.19  U=47 (P=0.364) |
| **Smoking**  **Yes**  **No**  **U(P) or t (P)** | **44**  **6** | 16.28 ± 19.83  12.77 ± 20.73  U=97.50 P=0.311) | 0.75 ± 0.68  1.50 ± 1.41  U=93.0 (P=0.258) | 0.83 ± 0.89  1.77 ± 1.17  U=68 (P=0.057) | 5.92 ± 8.76  0.73 ± 0.63  U=61 (P=0.033*) | 14.06 ± 4.23  15.15 ± 7.03  t=0.546(P=0.588) | 2.60 ± 2.69  2.07 ± 0.50  U=123.50(P=0.805) | 2.57 ± 3.76  1.41 ± 1.16  U=112.0 (P=0.570) | 17.32 ± 3.28  14.11 ± 6.10  U=81.50 (P=0.134) |
| **Family history**  **Negative**  **Positive**  **U(P) or t (P)** | **36**  **14** | 16.42 ± 19.81  14.41 ± 20.29  U=239.5(P=0.787) | 0.69 ± 0.57  1.22 ± 1.20  U=207 (P=0.331) | 0.88 ± 0.94  1.09 ± 1.05  U=219.50(P=0.483) | 6.14 ± 9.29  3.14 ± 5.07  U=185.5 (P=0.151) | 14.06 ± 4.46  14.53 ± 4.99  t=0.327(P=0.745) | 2.70 ± 2.81  2.13 ± 1.63  U=241.5 (P=0.821) | 3.04 ± 4.0  0.84 ± 0.96  U=118 (P=0.004*) | 16.88 ± 2.56  17.06 ± 6.03  U=249.50(P=0.957) |
| **Tumor size**  **<5**  **>5**  **U(P) or t (P)** | **16**  **34** | 17.69±18.63  15.0 ±20.48  U=223 (P=0.308) | 0.6101±0.844  0.943 ± 0.798  U=162(P=0.007*) | 0.824±1.075  0.996±0.916  U=212.5 (P=0.216) | 5.585±11.91  5.162±6.315  U=206.5 (P=0.173) | 14.38 ± 4.35  14.10 ± 4.73  t=0.204(P=0.839) | 2.193 ± 2.235  2.698 ± 2.671  U=230.5 (P=0.388) | 2.705 ± 5.245  2.296 ± 2.506  U=232 (P=0.405) | 17.59 ± 2.65  16.62 ± 4.22  U=217 (P=0.253) |
| **Lymph node metastasis**  **Negative**  **Positive**  **U(P) or t (P)** | **8**  **42** | 7.76 ± 14.38  17.40 ± 20.39  U=99.0 (P=0.069) | 0.90 ± 0.95  0.82 ± 0.81  U=168 (P=1.00) | 0.60 ± 0.61  1.0 ± 1.01  U=138.50(P=0.442) | 4.86 ± 6.09  5.38 ± 8.81  U=150.50(P=0.649) | 14.73 ± 2.80  14.09 ± 4.85  t=0.360(P=0.720) | 1.64 ± 1.44  2.71 ± 2.66  U=136 (P=0.412) | 2.72 ± 4.23  2.37 ± 3.48  U=157 (P=0.785) | 17.05 ± 1.09  16.91 ± 4.12  U=150.50(P=0.649) |
| **Stage**  **II +III**  **IV**  **U(P) or t (P)** | **10**  **40** | 15.29 ± 21.73  16 ± 19.52  U=169 (P=0.452) | 0.830 ± 0.864  0.838 ± 0.819  U=197 (P=0.942) | 0.805 ± 0.92  0.973 ± 0.99  U=189.5 (P=0.799) | 4.96 ± 5.78  5.38 ± 8.97  U=171.5 (P=0.489) | 16.03 ± 6.048  13.72 ± 4.085  t=1.442(P=0.156) | 2.29 ± 2.15  2.59 ± 2.63  U=199 (P=0.981) | 2.69 ± 3.73  2.36 ± 3.56  U=187 (P=0.753) | 16.7 ± 1.24  16.96 ± 4.20  U=197.5 (P=0.952) |
| **Grade**  **I+II**  **III**  **U(P) or t (P)** | **27**  **23** | 13.12±17.58  19.07 ± 22.0  U=245 (P=0.202) | 0.706 ± 0.623  0.989 ± 0.995  U=266 (P=0.386) | 1.021 ± 0.99  0.844 ± 0.93  U=272.5 (P=0.459) | 7.28 ± 10.21  2.964 ± 4.77  U=170.5(P=0.006*) | 14.39 ± 4.65  13.95 ± 4.57  t=0.334(P=0.740) | 3.30 ± 2.97  1.629 ± 1.48  U=180.5(P=0.011*) | 3.72 ± 4.39  0.904 ± 0.997  U=95.5(P=<0.001*) | 17.14 ± 2.13  16.67 ± 5.14  U=259.5 (P=0.321) |

The results were expressed mean ± S.D. U: Mann Whitney test t: Student t-test

P: P value for comparing between different categories.
